# The Role of Melanoma Cell-Derived Exosomes (MTEX) and Photodynamic Therapy (PDT) within a Tumor Microenvironment

**DOI:** 10.3390/ijms22189726

**Published:** 2021-09-08

**Authors:** Bridgette Mkhobongo, Rahul Chandran, Heidi Abrahamse

**Affiliations:** Laser Research Centre, Faculty of Health Sciences, University of Johannesburg, P.O. Box 17011, Doornfontein 2028, South Africa; bbmkhobongo@yahoo.com (B.M.); habrahamse@uj.ac.za (H.A.)

**Keywords:** melanoma, exosomes, extracellular vesicles (EVs), melanoma cell-derived exosomes (MTEX), tumor cell-derived exosomes (TEX), photodynamic therapy (PDT), photosensitizer (PS), reactive oxygen species (ROS)

## Abstract

Photodynamic Therapy (PDT), an unconventional cancer therapy with optimistic desirable effects, utilizes the delivery of a photosensitizer (PS) that is activated by light at a particular wavelength and inducing oxidative cytotoxic damage of a tumor and its surrounding vasculature. Deeper seated tumors such as internally metastasized melanomas are more difficult to treat with PDT as the penetration of laser light to those sites is less. Limitations in targeting melanomas can also be attributed to melanin pigments that hinder laser light from reaching targeted sites. Exosomes serve as naturally occurring nanoparticles that can be re-assembled with PSs, improving targeted cellular absorption of photosensitizing agents during PDT. Additionally, studies indicate that exosomes released from PDT-treated tumor cells play a critical role in mediating anti-tumor immune responses. This review collates the role of Melanoma Cell-Derived Exosomes (MTEX) in immune response mediation and metastasis. Tumor Cell-Derived Exosomes (TEX) post PDT treatment are also reviewed, as well as the effects of exosomes as carriers of photosensitizers and delivery systems for PDT. The understanding and research on the role of melanoma exosomes induced by Photodynamic Therapy and their tumor microenvironment will assist in future research in treatment prospects and implications.

## 1. Introduction

The prevalence of melanoma is on the rise and older population are mainly affected with an increased incidence later in life. The treatment is primarily dependent on the size and grade of melanoma. Treatment for advanced stages of melanoma is limited, and the overall benefits might be limited due to toxicities, as well as the development of diverse resistant mechanisms. The principal method of prevention for melanoma is mainly the application of sunscreen and frequent examinations of the skin to identify atypical moles [1].

Photodynamic Therapy is a recent emerging complementary and unconventional treatment method for tumors and may be a suitable palliative treatment option since it is a minimally invasive procedure. Treatment of tumor cells with PDT results in irreparable destruction of tumor cells and tumor-associated blood vessels. It is also responsible for triggering antitumor, immune, and inflammatory responses. PDT has been shown to treat nonmalignant and malignant diseases; however, melanoma defense systems such as pigmentation, and oxidative stress resistance are such limiting factors that can possibly reduce the efficacy of PDT [2].

Exosomes are the smallest subset of bilayer extracellular vesicles (EV) occurring in all body fluids, and are manufactured by all cells undergoing physiological and pathological conditions. They function as components that engage a crucial role in the intercellular communication network. Exosomes can be re-assembled and used as natural carrier and delivery platforms due to their nanoscale size, low toxicity, editable surface structure, structural stability, and cargo loading ability [3]. Exosomes that are re-assembled with PS have been reported to successfully deliver PS into target cells and generate the required, effective amount of reactive oxygen species (ROS) when exposed to laser treatment for photodynamic therapy [4]. Multiple techniques have been developed for the loading of desired therapeutic or diagnostic cargo (nucleic acids, peptides, proteins, nanomaterials, and dugs) into exosomes, conferring the targeting capacity of exosomes [3]. However, early and late onset side effects, including other limitations must be studied.

### 1.1. Melanoma Background

Melanoma is a malignant tumor or neoplasm of the melanocyte cells that are normally rooted in the deepest segments of the epidermis and produce the melanin pigment found in the skin. Although it is the least commonly found type of skin cancer, the melanoma incidence frequency has doubled. It is highly aggressive and is recognized as the prime source of death attributable to its metastatic behavior [1]. Melanocytes are triggered by ultraviolet radiation, which increase melanin pigment production and studies show that melanoma affects males and females differently. The National Cancer Institute in 2020 reported that while the risk for occurrence of melanoma may increase with age, it may occur at any age group. The median age at diagnosis has been reported to be 65 years. The 78th percentile of diagnosed patients is said to be 45 to 84 years of age [5]. The incidence of melanoma cases in the Ekurhuleni Population–Based Cancer Registry (EPBCR) in South Africa has been recorded by the National Cancer Registry in 2018. Statistics from this registry indicated a higher prevalence of melanoma in White males, with a recorded frequency of 62. Black males had a recorded frequency of nine, while Colored and Asian males had a frequency of one each. The prevalence in White females, Black females, and Colored females was recorded to have a frequency of 35, 5 and 2, respectively [6].

Although melanoma may arise on any superficial skin surface, it is mostly predisposed to grow on already existing moles. In males, it tends to occur on the upper body parts usually exposed and damaged by the sun such as the head, neck, and anywhere across the shoulders and hips. Common sites of occurrence for females may lie in between the shoulders and between hips. Melanoma is staged into five, wherein stage 0, the melanoma is in situ and only spreads radially on the skins’ surface. It is only detected in the most superficial skin layers while still having the basal lamina undamaged. In stage I, the tumor spreads both radially and vertically, but still within 1 mm of thickness and protrudes in the direction of the basil lamina expanding into the papillary dermis. In stage II, the tumor lies within 1 and 2 mm thick and extends vertically, while growing more rapidly into the papillary-reticular dermis interface. In stage III, the tumor develops vertically into the reticular dermis and the melanoma cells protrudes into a minimum of one lymph node or towards surrounding tissue. In stage IV, the cancer cells continue to protrude radially and vertically into the subcutaneous tissue. The melanoma also metastasizes to other organs by entering blood vessels or through lymphatic vessels either to the lungs, other skin areas, or lymph nodes that are distant from the initial expansion [7].

Risk factors constituting melanoma occurrence entail excessive subjection to ultraviolet (UV) radiation. Melanomas are extremely prevalent in fair-skinned individuals while being far less frequent in individuals with dark skin. Severe blistering sunburns, prolonged duration of sun exposure, dwelling in elevated altitudes that experience the sun rays at a much harsher level, and a weakened immune system also comprise risk factors. Increased incidences of melanoma are seen in individuals with previous melanoma, squamous cell or basal cell skin cancer diagnosis are more likely to develop an additional form of melanoma. A family history of melanoma sets individuals at an increased risk, where 8–12% instances of cutaneous melanoma are inherited [8]. Other risk factors that institute the development of melanoma include viruses and chemicals.

### 1.2. Current Melanoma Treatment Modalities

Various factors contribute to treatment options for individuals. These factors not only include the stage of the melanoma and locations, but also the depth, its spread, the rate of growth, number of metastases, specific genetic changes, the patient’s general health, age, preferences, and other medical conditions [1].

Current approved conventional treatment options include surgical removal, radiation therapy, targeted therapy, immunotherapy, or chemotherapy if there is a chance for cancer re-occurrence. Cases that have metastasized (stage IV) are usually difficult to surgically remove and are termed unresectable. Intra-tumor heterogeneity, which involves the diverse subpopulations within and between tumor lesions that are associated, affects the response of the tumor to drug therapy in melanomas profoundly. Significant results have been observed previously from targeted therapy that aims at stunting a tumor’s survival pathway and its mechanisms for proliferation, within a cancer cell. Although this has been evident for various solid tumor types, targeted therapy does not eradicate cancer in all molecularly selected patients with advanced cancer stages even if short-lived clinical benefits are witnessed [9,10].

The treatment of stage 0 melanoma (in situ) may usually be treated with surgical removal, incorporating wide excision that removes the primary melanoma and its normal bordering tissue. In addition to stage I surgical excision methods, Stage II may also include lymph node mapping and removal, followed by treatment with interferon if chances of recurrence are suspected. Resectable stage III melanoma treatment incorporates surgical excision and lymph node mapping, which helps determine the need for lymph node dissection should the tumor be located there. To eliminate cancer recurrence in stage III melanoma, surgery is usually accompanied by immune checkpoint inhibitors such as nivolumab, pembrolizumab, or ipilimumab for immunotherapy. Targeted treatment with signal transduction inhibitors such as trametinib and dabrafenib is also an option for follow-up treatment. Immunotherapy clinical trials including or excluding vaccine therapy, or a clinical trial of therapies that are selective to distinct gene alterations, can also be used as alternative treatment options when other options are ineffective. Stage IV melanoma is challenging to eradicate and in most cases cannot be surgically removed. Currently approved treatment options are chemotherapy; oncolytic virus therapy with talimogene laherparepvec, which is injected into the tumor; immunotherapy with interleukin-2 (IL)-2 pembrolizumab, ipilimumab, nivolumab, or ipilimumab (nivolumab sometimes given conjointly); targeted therapy with signal transduction inhibitors such as binimetinib, cobimetinib, dabrafenib, encorafenib, trametinib, or vemurafenib (also sometimes given conjointly); and palliative therapy that aims to better the patients’ quality of life [8].

Tumors are heterogeneous and encompass various dynamic subpopulations of cells, of these being cancer stem cells (CSC). Cancer stem cells possess the potential to bring about cancer relapse and metastasis and are accounted as being resistant to multiple forms of therapy [11]. Current forms of cancer treatment may successfully destroy cancer cells; however, are ineffective at eradicating resistant cancer stem cells. It is imperative to find therapies that can eliminate cancer stem cells and it has become a major concern. The implications of intra-tumor heterogeneity, including cancer stem cells, compel the obligation of research on improved treatment interventions and alternative methods for metastatic melanoma and cancer stem cell treatment. Photodynamic therapy is a propitious alternative form of unconventional cancer treatment. The benefits of photodynamic therapy involve minimal side effects, and it is a non-invasive procedure that can be carried out repeatedly, often costing less when compared to other forms of conventional treatments [12].

### 1.3. Photodynamic Therapy Mechanism of Action

**Figure 1:**
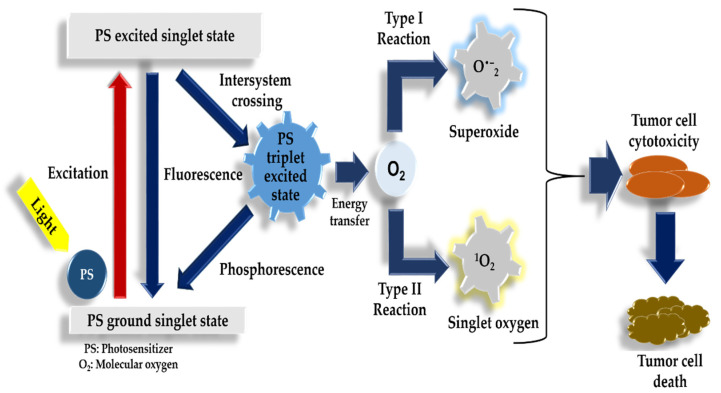
Photodynamic therapy mechanism of action – Type I reaction: generation of superoxide radicals and Type II reaction: generation of singlet oxygen. Generated ROS inducing tumor cell death.

Photodynamic therapy uses the combination of a photosensitizer, intracellular molecular oxygen within tissue, and light for the destruction of cancer cells. Post administration, the photosensitizer accumulates in the targeted tissue. Once the area is exposed to localized irradiation by the emission of fluorescence light, the creation of extremely toxic ROS limited to that area will be prompted, as seen in Figure 1. These characteristics enable imaging, which is known as photodetection. It also allows for irradiation, known as photodynamic therapy of the cancerous tissue. This involves the mechanism of action that takes place in complete oxygen-deprived (anoxic) environments. The light initiates the excitation of the photosensitizer, which promotes an electron to an elevated energy state [13]. Another mechanism of action is driven by the excitation of the photosensitizer by light. The energy is transmitted to the ground state of the molecular oxygen resulting in the excitation of singlet oxygen species, which go on to impair cellular functions resulting in tumor destruction [14].

The oxygen and photosensitizer interact through the triplet states due to oxygens’ distinct triplet-ground state features and being excitable in low-lying energy states. The energy prerequisite to produce singlet oxygen is comparatively low when compared to that needed for the triplet to singlet transition. The energy needed in oxygen has been recorded as 22 kcal mol-1. This recorded energy correlates to the energy of a wavelength of 1274 nm at infrared light [15]. According to Figge et al., these reactions that are dependent on oxygen concentrations are known as type II photoreactions and are established as dominating during photodynamic therapy [16].

### 1.4. Exosomes and Extracellular Vesicles

**Figure 2:**
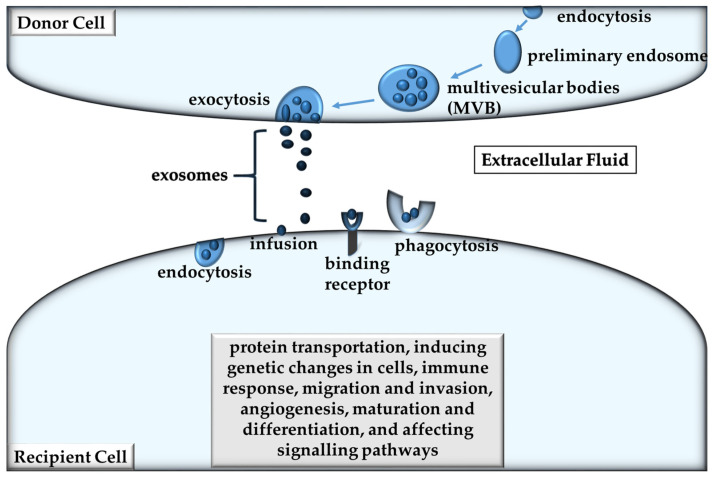
Exosome Biogenesis Process—Endocytes form premature endocytes, which lead to late endocytes or multivesicular bodies (MVBs). Later, exosomes are then released through exocytosis. Exosomes effects on the recipient cell are: protein transportation, inducing genetic changes in cells, immune response, migration and invasion, angiogenesis, maturation and differentiation, and affecting signaling pathways.

Exosomes partake in a crucial role within a tumor microenvironment and are delineated as being extracellular vesicles (EVs) [17,18]. They are approximately 30–150 nm in diameter and are the smallest of the EVs. All cell types release them and they form part of the mediation of intercellular communication [19]. The origin-unique cargo of exosomes makes them unique from other EVs as they have the ability to epitomize the molecular attributes of their cell of origin during their biogenesis process in an endosomal compartment [20]. The exosome biogenesis process can be seen in Figure 2, where inward budding of the endosomal membrane creates multivesicular bodies (MVB), whereupon they amalgamate with the plasma membrane, forming exosomes [21]. The generation of EVs advances from cavitations in endosomes that are expelled when the plasma membrane fuses with multivesicular bodies (MVBs) [22]. Thereafter, they are diffused by means of lymphatic system or the circulatory system into local tissue, as seen in Figure 2 [21]. These vesicles enclose proteins and nucleic acids and are safeguarded from degradation as they resist proteolytic and nuclease activity [23]. Proteins, deoxyribonucleic acid (DNA), messenger ribonucleic acid (mRNA), micro ribonucleic acid (miRNA), and lipids designate the molecular constituents of exosomes [24].

Exosomes participate in biological function mediation such as intracellular communication, metastasis and drug resistance. In addition to this, exosomes are capable of facilitating protein transportation, inducing genetic changes in cells, maturation and differentiation, and affecting signaling pathways [17,18]. They are categorized into subtypes that include characterization by size, cell origin, biochemical composition, or description of cell conditions [25]. Some of the procedures for deriving and refining exosomes from biological fluids or cell culture media comprise a process called gradient centrifugation. Released EVs have a phosphatidylserine cell membrane translocating from the inner to the outer leaflet, making it stain positive for annexin-5 [26].

The characteristics and composition of melanoma exosomes exhibit the constituents of the donor cell, established on the physiological conditions the cell is encountering [27]. Various molecular mechanisms are accountable for the selective classification of exosomes from parental cells and result in the heterogeneity of exosomal content seen [28]. Differences and similarities between melanoma exosomes and neoplastic exosomes of other cancer types exist [29,30].

Melanoma exosomes are normally secreted by keratinocytes, which increase the expression and activity of melanosome proteins by interacting with melanocytes [31]. The membrane composition of melanoma exosomes consists of lipids, proteins, and ribonucleic acid (RNA) that are the same as some of the parent membrane cell [32]. Lyso-phophatidyethanolamine, sphingomyelin, lyso-phosphatidylcholin, and cholesterol are enriched raft-like domains that exist on numerous cancer exosomes [33].

The melanoma exosome RNA is also similar to that of the parent cell and the role of these RNAs tend to be involved in approaches that promote tumor progression as opposed to tumor suppressing roles [34]. The exosomes extricated from A375 melanoma cells when compared to ordinary melanocytes show enhanced levels of miRNAs, such as lethal 7a (let-7a), miR-30b, miR-30d, miR-15b, miR-182, miR-221, miR-222, miR-31, miR-19b-2, miR-20b, miR-92a-2, miR-21, miR-532-5p, and miR-210 [35]. The proteins in melanoma exosomes differ from that of normal melanocytes. Tumor marker proteins such as isoform of heat shock protein 70 (Hsp-70) are distinct to melanoma and are suggestive of increased tumor progression [36]. Several other proteins like tyrosinase-related protein 2 (TYRP2), very late antige-4 (VLA-4), Hsp-70, major histocompatibility complex (MHC) I, melanoma antigen recognized by T cells 1 (Mart-1), mitogen-activated protein kinase kinase kinase kinase (MAPK4K), from human epidermal growth factor receptor 2 (Her2/neu), transient receptor potential (TRP), cluster of differentiation (CD)44, guanosne-5′-triphosphate (GTP)-binding proteins, annexin A2, disintegrin and metalloproteinase domain-containing protein 10 (ADAM10), and glycoprotein 100 (GP100) are also seen enhanced in exosomes acquired from malignant melanoma, possibly serving as prognostic biomarkers [33,37,38]. Ribosomal proteins like RPL4, RPL35, RPL19, RPS11, RPL13, and RPL5, have an overexpression in melanoma exosomes [39].

## 2. The Role of Exosomes in Immune Responses

Immunologically active exosomes are discharged from both tumor and immune cells. They both alter the functions of immune cells, subject to the stimulatory or suppressive protein cargo ratios they carry and subsequently determine immune-inhibitory functions. These exosomes affect immune surveillance, intercellular communication, activation of immune cells, and antigen expression. In Table 1, the inhibition of immune cell proliferation, apoptotic induction of activated CD8+ T-cells, suppression of natural killer (NK) cell activity, interference with monocyte differentiation, and the promotion of regulatory T-cells (Treg), including the expansion of myeloid-derived suppressor cells (MDSC) are among the effects of tumor-derived exosomes (TEX) on immune cells. Furthermore, exosomes derived from tumor cells may also participate in the emergence of cancer therapy drug resistance [40].

Exosomes existing in the plasma are heterogeneous and constitute a composition that includes melanoma cell-derived exosomes (MTEX) and non-melanoma cell-derived exosomes (non-MTEX). Exosomes have a dual potential in modulating the response of tumors to therapy. This is achieved through regulation of the immune system that is exosome-mediated. MTEX impede the processing of human primary immune cells as they transfer an abundance of immunosuppressive proteins. In contrast to this, non-melanoma cell-derived exosomes (non-MTEX) prompt immune cell activity [49,50]. The production of exosomes is higher in melanoma tumor cells than that of normal melanocytes. The plasma of melanoma patients harbor increased levels of exosomes that contain immunosuppressive proteins such as Fas ligand (FasL) and transforming growth factor beta (TGF-β), melanoma-associated antigens (MAAs), and oncoproteins, including Myc [18,47,51,52].

**Figure 3:**
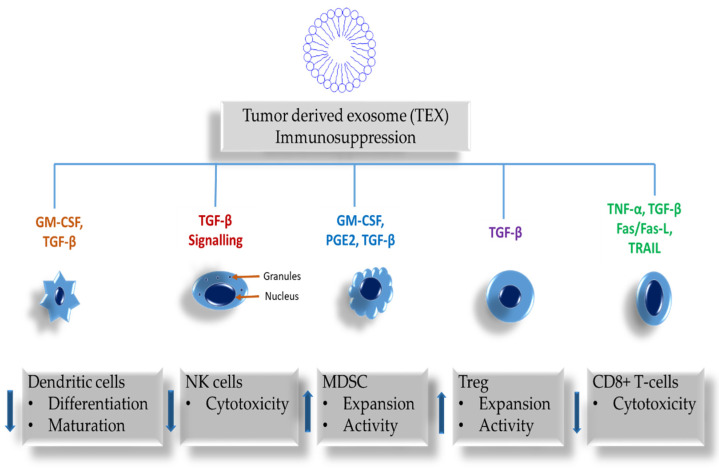
The effects of tumor-derived exosomes (TEX) on immune cells—Inhibition of immune cell proliferation (down arrows indicates down-regulation and up arrows indicates up-regulation): Apoptotic induction of activated CD8+ T-cells, induced by up-regulated TGF-β or by inhibitory signals. Suppression of NK cell activity through exosomal TGF-β and toll-like receptor (TLR) activation. Interference with monocyte differentiation and the promotion of Treg, including the expansion of MDSC.

In vitro studies of exosomes attained from melanoma cell line supernatants showed primary human cell function inhibition and clinically that melanoma patients’ plasmas were immunosuppressive [47,51,53]. Disease progression in melanoma due to immune suppression caused by tumor escape from the immune host system is a common presentation [54]. In melanoma, the mechanisms managing tumor-induced immune suppression encompass programmed death-ligand 1 (PD-L1) excessive expression in the tumor, and programmed death-ligand 1 positive (PD-L1+) tumor-derived exosomes. These have been declared as having a role with the interference of a patients’ responses to oncological immunotherapies [53,55]. A study done by Theodoraki et al. contrasted the strong suppression of T cell activity by exosomes with increased PD-L1 levels; and the non-suppression with low levels of PD-L1 [56]. Tumor cell-derived exosomes (TEX) interact with immune cells and deliver negative signals, impeding their anti-tumor functions, as depicted in Figure 3. Direct or indirect immunosuppressive molecules transported by TEX have an impact on the development, maturation, and anti-tumor undertakings of immune cells [57]. Tumor progression and tumor escape may be facilitated by TEX that originate from donor cells that are actively engaged in the suppression of antitumor immunity, including suppressing effector cell functions [58].

Dendritic cell-derived exosomes (DEXs) regulate immune operations either through the MHC class I and/or II proteins that are presented on exosomes or by the transmission of their contents to other cells [59]. When compared to immature dendritic cells, DEX can induce T-cell stimulation and promote CD8+ T-cell activation [60]. The melanoma exosome mediated modulation of mitochondrial respiration enables the alteration of the transcriptome of CD8+ T-cells, and the induction and activation of pro-tumor macrophage polarization [61,62]. The presentation and secretion of miR690 and Rab27a by melanoma exosomes also activates CD4+ T-cells [63].

## 3. Role of Exosomes in Metastasis

Exosomes play a role in the metastatic progression of tumors. Primary tumor and remote tissue are both modulated by exosomes to promote metastasis. This is enabled by the capacity of exosomes to bear and transfer proteins and nucleic acids [48]. The transfer of this metastatic ability or invasive nature of cells to local cells and remote cells instructs and assists in creating a pre-metastatic niche that is favorable for tumor formation at the site [64]. This possibly promotes cell recruitment and migration. Exosomes released from cancer, when subjected to hypoxic conditions contain enhanced TGF-β, tumor necrosis factor alpha (TNF-α), and IL-6 cytokines levels. These particular cytokines significantly partake in the promotion of metastasis [65]. Other conditions of applied stress such as oxidative stress, heat, and cryostasis result in the production and release of a higher amount of exosomes by melanoma cells when compared to cells that are not subjected to stress [66].

Melanoma-derived exosomes contribute as message-bearers, promoting tumor metastatic invasion. Some of the proceedings accountable for metastasis are MTEX enhanced with oncoproteins and are released into the extracellular milieu. Such exosomes form a pre-metastatic niche by remodeling the extracellular matrix, promoting proangiogenic events, inducing inflammation, and increasing vascular permeability. This allows for migration to other sites such as the lymph nodes, lungs, and bone marrow. Melanoma exosomes are responsible for driving the invasive capacity of tumor cells. An increased expression of matrix metalloproteinases like a-disintegrin-and-metalloproteinase (ADAM) and a-disintegrin-and-metalloproteinase with thrombospondin-motifs (ADAMTS) can be found when examining the proteomics of TEX. The lytic metalloproteinases partake in alleviating a cancers’ invasion potential by breaking down the extracellular matrix of a cancer cell [24].

Melanoma exosomes also mediate tumor angiogenesis. A study done by Hood et al. demonstrated the angiogenesis promotion by exosomes that is achieved by prompting the formation of endothelial spheroids [67]. Studies indicate that melanoma cells with an increased expression of Wnt5a induce an instantaneous extrication of exosomes that contain immunomodulatory cytokines IL-6 and pro-angiogenic factors IL-8, matrix metallopeptidase 2 (MMP2), and vascular endothelial growth factor (VEGF) in their cargo [68]. A major role is played by an increase in angiogenic factors like IL-8, VEGF, platelet-derived growth factor (PDGF), fibroblast growth factor (FGF), and TGF. These factors stimulate angiogenesis and the regulation of migration, the proliferation of endothelial cells, and the networking of quiescence [69]. In another study, angiogenesis and metastasis were also promoted by the initiation of JAK-STAT pathway, which was all regulated by MTEX that contained miRNA-9 and were freely taken up by endothelial cells [70].

Melanoma cells also mediate cancer lymph node metastasis. The role of melanoma cell gene expression in melanoma metastasis to lymph nodes has been studied [71,72]. Three critical deductions made from three genes that were upregulated due to exosome treatment were: cell recruitment proteins that promote lymph node migration, extracellular matrix proteins, and vascular growth factors. Other effects mediated by melanoma exosomes include cell differentiation.

## 4. Exosomes as Carriers and Delivery Systems for PDT

**Figure 4:**
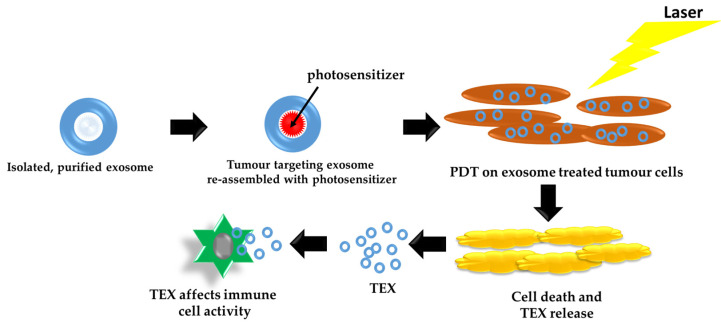
Re-assembled exosome as a drug delivery carrier loaded with a photosensitizer. TEX affects the activity of immune cells.

Frequently, the response to conventional therapies is low in patients with metastatic or recurrent melanoma, with some cases developing into drug resistance. Increasing the effectiveness of treatments, preventing drug resistance, and decreasing cytotoxic side effects might be alleviated by targeted delivery of therapeutics. Exosomes, responsible for intracellular communication, display a high affinity for cellular uptake by original cells. Studies have shown that exosomes have a high targeting specificity capacity and re-assembled exosomes retain their ability to target parent cells, as depicted in Figure 4.

Exosomes possess a high affinity for specific targeting and numerous pieces of research have ventured to establish modified exosomes loaded with drugs and use them as exosome-based drug delivery carriers [73,74,75,76,77,78]. In their study, Jang Y et al. made use of a modified tumor-derived re-assembled exosome (R-Exo) as a drug delivery carrier loaded with a chlorine e6 (Ce6) photosensitizer, which had concurrent photosensitizer and immuno-stimulatory attributes. After this modification, the Ce6-R-Exo photosensitizer enhanced the secretion of cytokines from immune cells, progressing to the ability of use as an immunotherapeutic agent [78]. The high-targeting exosomes successfully delivered the Ce6 photosensitizer into target cells and generated the required, effective amount of ROS when exposed to laser treatment for photodynamic therapy [4].

Some of the qualities that make exosomes favorable and ideal carries for macromolecules, especially oligonucleotides-like small interfering ribonucleic acid (siRNA), are that they are biocompatible and have negligible bio-toxicity due to the possession of a structure and composition that is similar to the cytomembrane. Additionally, exosomes escape immuno-surveillance and infiltrate deep into tissues, delivering therapeutic drugs directly to cells [79]. Remarkably, exosomes have the capacity to track target cells or organs unique to them [72]. Other beneficial features that exosomes encompass are high drug-loading capacity, proficiency in directed or sustained cargo secretion, unique tissue-targeting, and protein defense against proteases.

Exosome loading techniques with siRNA or miRNA have been designed and developed. Examples of non-specific methods are electroporation, passive diffusion of components, and transfection techniques [80,81]. Targeted exosome-based nanoparticles are frequently based on the strategy of incorporating the use of genetically engineered cell lines that express fusion proteins. This strategy largely consists of an extra-vesicle transmembrane domain and a targeting domain. One such study done by Yang et al. is an example. In this study, a multifunctional chimeric peptide (ChiP)-modified exosome was developed for nucleus-targeted PDT. The ChiP-modified exosome constituents were a peptide (PKKKRKV) for nuclear translocation, a protoporphyrin IX (PpIX), which is a photosensitive drug for PDT, and a C16 alkyl chain for exosome modification. This resulted in noteworthy tumor repression in both in vivo and in vitro studies [82].

## 5. Effects of Tumor Cell-Derived Exosomes (TEX) Induced by PDT

Melanoma cell-derived exosomes (MTEX) confer changes to surrounding cells via transfer of their cargo, shifting the way cells respond to therapy. Examining the in vivo and in vitro Melanoma cell microenvironment response post PDT and other forms of therapy is crucial. Exploring the role of vesicle release and dissemination in melanoma due to treatment, including PDT will possibly improve the effects of PDT and better the long-term survival of melanoma patients. Photodynamic Therapy is capable of causing a release of a huge amount of EVs from melanoma cells after PDT exposure.

Generally, exosomes are manufactured and released by all mammalian cell types that are either diseased or healthy in the body [81]. Studies and reports by Pitson et al., Bae et al., Yu et al., and Kucharzewska et al. have suggested that exosomes found in cancer cells generally have a different biogenesis and exosome trail. They report that cells that are exposed to stress, exhibit elevated exosome discharge [83,84,85,86,87]. Thery et al. explain in their article that the expulsion of extracellular vesicles might be prompted by cell initiation or stress [88]. Gross manufacture and excretion of extracellular vesicles is one of the responses to photodynamic therapy of in vivo and in vitro cells [89]. Table 2 describes the outcomes of exosomes derived from PDT-treated tumor cell lines. Post-PDT exosomes are seen to be involved in exosome-mediated reprogramming. In their study, Zhao et al. (2020) report findings of exosomes capable of bringing about elevated anti-tumor immune response through stimulating fibroblast to expel TGF-β1 and the maturation of DC. These exosomes where attained from 5-aminolevulinic acid (ALA) PDT-treated squamous cell carcinoma (SCC), tabulated in Table 2 [90]. Theodoraki et al. demonstrated in their clinical study that exosomes found in the plasma of patients with neck and head squamous cell carcinoma (HNSCC) who are undergoing PDT, have the ability to reprogram tumor cells toward an epithelial phenotype. In addition to this, post-PDT exosomes were reported to carry epithelial cadherin (E-cadherin), restore epithelial cell adhesion molecule (EpCAM) expression in tumor cells, and epithelial morphology. Other significant changes were the down regulation of mesenchymal gene expression, inhibited proliferation, as well as inhibited migration and invasion [91]. Moreover, exosomes may aid as unrealized biomarkers of therapy response prognosis, and possibly diagnosis, due to their unique tumor or immune cell-derived molecular contents that allow for the alteration of the recipient cell functions.

In their study, Aubertin et al. supports the hypothesis that administration of mild PDT induces apoptosis that is reversible, accompanied by a large quantity of extracellular vesicles from cancer cells. Also supporting the continuation of the hypothesis saying the administration of a stronger photosensitizer prompts irreversible damage such as tumor cell death perhaps through necrosis, and is accompanied by less vesicle release [90]. The data from this study suggests that profuse EV secretion prompted by non-severe cytotoxic therapy has the potential of exacerbating tumor conditions in cancer patients. In addition, other forms of stress exerted on cancer cells include the starvation of cells; however, the effect of this is a far less vesicle excretion than the effects due to PDT. Furthermore, cells undergoing stress due to chemotherapeutic cell damage, using the drug Doxorubicin (DOX) induced far less vesicle release than PDT [90].

According to Aubertin et al., the spread of oncogenes and oncoproteins might be caused by the intercommunication from the extrication of extracellular vesicles between cancer cells and naïve neighboring as well as distant cells. This might also cause drug resistance at remote sites through drug transferal. Other components that might be transferred include extracellular membrane components and large intracellular components. Anticancer treatments are thus possibly capable of spreading oncogenes and oncoproteins to other remote sites, possibly causing tumor initiation and drug resistance [90].

## 6. Clinical Trials Using Exosomes and Photodynamic Therapy in Melanoma Therapy

Clinical trials on exosomes for use as drug delivery vehicles, biomarkers, or biological targets to treat a wide variety of human disease conditions are being explored [92]. Up to date, however, no clinical trials have been reported for PDT using exosome engineered photosensitizers in the treatment of melanoma. A clinical trial to stimulate the immune response in stage IIIb/IV133 Melanoma patients was done by Escudier et al. The clinical trial involved the use of exosomes derived from the DCs of each patient. The exosomes were modified to present tumor antigens and were reinjected into the same patients. Outcomes included an increase in the quantity of NK cells, increased expression of NKG2D in CD8+ and NK cells, no major (>grade II) toxicity, and a lack of specific CD8+ response. [93].

## 7. Pros and Cons of Photodynamic Therapy and Exosomes in Cancer Therapy

Some of the advantageous attributes of re-assembled exosomes are that they retained their ability to target their original donor cells, retaining the same morphology as the lipid bilayer of naïve exosomes. They possess cellular uptake efficiency and high intercellular uptake efficiency, while effectively delivering more photosensitizers to the cells with high targeting ability generating enough ROS under laser exposure for effective photodynamic therapy. It is also reported that exosome-based photosensitizer delivery systems also maintain reduced damage to other tissues [78]. Due to their size, exosomes diffuse passively into tumors through the enhanced permeability effect (EPR) escaping from the mononuclear phagocyte system (MPS) [82]. They have high flexibility and compatibility, with capacity to cross various biological/physical barriers including the blood–brain barrier [94] and can be administrated intravenously, intranasally, intraperitoneally, and intracranially [95].

Disadvantages of exosomes-based delivery systems include complex procedures of multifunctional modifications that limit biomedical applications due to poor drug cargo loading efficiency. Various cells yield different exosome secretion capacities. Low efficient industrial production methods for exosome isolation and purification are time consuming, costly, and involve large-scale cell culturing [96].

## 8. Toxicological Issues in Photodynamic Therapy

Photodynamic therapy is generally considered to have minimal side effects and is a non-invasive form of treatment. Viable evidence of cumulative toxicity or photocarcinogenic risk has not been reported. Outcomes of PDT not only rely on concentration defined molecular targets, but also on the different cell types, different photosensitizers, different incubation times, and illumination conditions. Acute adverse effects such as pain can be diminished through adjusting low irradiation PDT procedures without compromising successful treatment delivery. In comparison to normal cells, photosensitizers tend to accumulate in significantly higher concentrations in tumor cells [97]. Some characteristics of a good PS that limit the occurrence of possible toxicological effects include the use of a PS that prevents possible photosensitivity caused by sunlight by having an absorption range of 400 to 600 nm. Overlapping of PS absorption bands with absorption bands of other substances in the body should be avoided [98].

Early onset side effects observed during or within hours or days of PDT have been reported as local skin reactions and pain. Rare side effects that occur are contact dermatitis, or erosive pustular dermatosis of the scalp and urticaria. Noteworthy effects on the immune system are documented where PDT either stimulated or, in some instances, repressed innate and adaptive immune responses. Late onset side effects of PDT although rare, are induced hyperpigmentation and scarring, Bullous Pemphigoid, and have the potential to induce or stimulate skin carcinogenesis [99].

## 9. Conclusions

Current forms of cancer treatment may successfully destroy cancer cells; however, are ineffective at eradicating resistant cancer cell populations and their stem cells. It is imperative to find therapies that can eliminate cancer stem cells that are the helm for cancer recurrence and metastasis. Metastasized melanomas are more difficult to reach with laser light. Furthermore, their melanin pigment responds poorly to photodynamic therapy. It is not only vital, but also beneficial to utilize photodynamic therapy components that maximize the level of photosensitizer absorption. Absorption can be achieved through targeting tumor cells and administering precise laser wavelengths and effective exposure times to achieve the highest excitation of effective photosensitizers. Although melanoma exosomes possess distinct qualities, numerous cancer types share the same tumor growth and metastasis promoting mechanisms, the effects of PDT-induced exosomes utilizing various photosensitizers in targeting melanoma cells and their stem cell population as well as their tumor microenvironment need further research. An approach directed at expanding the knowledge of photodynamic therapy effects of the tumor microenvironment and improving it as a cancer treatment through conducting more in vitro and in vivo experiments will create a better understanding.

Exosomes possess a significant role in mediating tumor disease progression and metastasis. Although melanoma exosomes possess distinct qualities, numerous cancer types share the same tumor growth and metastasis-promoting mechanisms. Recent research is directed towards comprehensively analyzing the role of exosomes in cancer progression. Aspects such as how the exosome/EV release, drug therapeutic concentration, and target cell damage of a given therapeutic approach interrelate should be considered. The biologically active contents of exosomes exhibit the changing molecular and genetic constituents of the PDT response of tumor parent cell. Furthermore, they are apt in inducing mRNA and protein adjustments in the tumor cell receiver. Studies have revealed that the phenotypic and functional modifications guided by plasma-derived exosomes might encourage or inhibit epithelial-mesenchymal transition in cells on the receiving end [91]. Exosomes may serve as unrealized biomarkers of therapy response prognosis, and possibly diagnosis, due to their unique tumor or immune cell-derived molecular contents that allow the alteration of the recipient cell functions.

## Figures and Tables

**Table 1:** Role of tumor-derived exosomes (TEX) in immunosuppression.

Immune Cells	TEX Effects	Non-TEX Effects	References
Natural killer (NK) cells	Suppressed cytolytic activity. Downregulation of NKG2D and impaired cell cytotoxic function.	Activating NKG2D, NKP30, NKP46, and NKG2C receptors inducing NK cell cytotoxicity	[41,42]
Dendritic cells (DC)	Suppressed differentiation, maturation, and activity. Suppress migration through chemokine receptor expression inhibition.		[43,44,45]
Myeloid-derived suppressor cells (MDSC)	Increase the progression of MDSCs, expediting their activation, aiding expansion, and increasing the immunosuppressive role.		[46]
Regulatory T-cells (Treg)	Increased proliferation and expansion. Induced immune suppression by supporting extension of CD4(+)CD25(+)FOXP3(+)T regulatory cells and the diminishing of antitumor CD8(+) effector T-cells.		[47]
CD8+T-cells	Suppressed cytotoxicity, inhibiting signaling and proliferation, increased apoptosis, and alteration of antitumor phenotype.	Promote the proliferation of all T-cells	[47,48]

**Table 2:** Effects of exosome induced by various photodynamic therapy on different tumor cell lines, using different photosensitizers.

Photosensitizer (PS)	Tumor Cell Line	Exosome Effects	References
Foscan^®^ m-THPC (5,10,15,20-tetra(3-hydroxyphenyl)chlorin) photosensitizer	Human prostatic cancer cells (PC-3, ATCC ^®^ CRL-1435™)	Increased EV release 1-h post photo-activation, secreted EVs may transmit extracellular membrane constituents, drugs and large intracellular objects to harmless target cells	[89]
(5,10,15,20-tetra(3-hydroxyphenyl)chlorin)	SCC cells (PECA, primary mice SCCs, A431)	ALA-PDT-treated SCCs trigger dendritic cell maturation and fibroblast secretion of TGF-β1, resulting in increased anti-tumor immunity.	[90]
Temoporfin (Foscan^®^ Biolitec, Vienna, Austria)	PCI13, a HNSCC HPV-negative cell line and SCC-90, a HNSCC HPV+ cell line; A549 (human lung adenocarcinoma), BT549 (human breast ductal carcinoma), and MDAMB231 (human breast adenocarcinoma)	Post-PDT exosome-mediated conversion from mesenchymal to epithelial tumor phenotype mediated by exosomes.	[91]

## Data Availability

Not applicable.
